# Schnitzler-Like Syndrome Presenting With IgG Kappa Monoclonal Gammopathy: A Case Report and Review of Diagnostic and Therapeutic Challenges

**DOI:** 10.7759/cureus.64440

**Published:** 2024-07-12

**Authors:** Gurjot Singh, Kanishka Goswami, Shubam Trehan, Meet P Kachhadia, Amna Farooq, Piyush Puri, Waqas Azhar

**Affiliations:** 1 Internal Medicine, Southern Illinois University School of Medicine, Springfield, USA; 2 Internal Medicine, Memorial Medical Center, Springfield, USA; 3 Internal Medicine, Saint John Hospital, Springfield, USA; 4 Hospital Medicine, Springfield Clinic, Springfield, USA

**Keywords:** igg kappa, joint pain, autoimmune disease, systemic inflammation, case report, chronic urticarial rash, autoinflammatory disorder, monoclonal gammopathy, schnitzler-like syndrome, schnitzler syndrome

## Abstract

Schnitzler syndrome (SS) is a rare autoinflammatory disorder characterized by a constellation of symptoms that include chronic urticarial rash, recurrent fever, arthralgias/arthritis, and monoclonal gammopathy, typically involving immunoglobulin M (IgM). However, cases with overlapping clinical features but lacking specific criteria fall under the umbrella of Schnitzler-like syndromes.

This case report describes a 40-year-old male with Schnitzer-like syndrome and underscores the diagnostic complexities and therapeutic challenges of Schnitzer-like syndrome with IgG kappa monoclonal gammopathy, highlighting the need for a comprehensive diagnostic approach and targeted therapy.

## Introduction

Schnitzler syndrome, first described by Dr. Liliane Schnitzler in 1972, is a rare autoinflammatory disorder marked by a constellation of symptoms, including chronic urticarial rash, intermittent fever, arthralgia, bone pain, and a monoclonal gammopathy, typically of the IgM type [1]. The syndrome bridges the gap between monoclonal gammopathies and autoinflammatory diseases, involving dysregulation of the innate immune system and aberrant cytokine signaling, particularly interleukin-1 (IL-1) [2].

The hallmark of Schnitzler syndrome is the presence of a monoclonal IgM protein in the serum, although variants presenting with IgG monoclonal gammopathy have also been documented [3]. These IgG variants, sometimes referred to as Schnitzler-like syndrome, expand the clinical spectrum of the disease and introduce significant diagnostic challenges. The exact prevalence of Schnitzler syndrome is unknown, but it is considered very rare, with fewer than 300 cases reported in the literature. The pathogenesis of Schnitzler syndrome is not fully understood, but it is believed to involve a complex interplay between genetic predisposition and environmental triggers that lead to an overactive immune response [4,5].

Monoclonal gammopathies involve the clonal proliferation of plasma cells that produce a single type of immunoglobulin. In classic Schnitzler syndrome, this manifests as an IgM monoclonal protein. However, cases with IgG or even absent monoclonal gammopathy are increasingly recognized as Schnitzler-like syndrome [6, 7]. This variant necessitates a comprehensive diagnostic approach due to its overlap with other autoinflammatory and hematologic disorders, such as adult-onset Still's disease, cryopyrin-associated periodic syndromes, and monoclonal gammopathy of undetermined significance (MGUS) [8].

The clinical presentation of Schnitzler syndrome can be highly variable, and the diagnosis is often delayed due to its rarity and the nonspecific nature of its symptoms. Patients typically present with a combination of fever, rash, and musculoskeletal pain, which can mimic a wide range of other conditions. Laboratory findings commonly include elevated inflammatory markers such as erythrocyte sedimentation rate (ESR) and C-reactive protein (CRP), and the presence of monoclonal gammopathy is a key diagnostic criterion [9].

The treatment of Schnitzler syndrome primarily involves targeting the IL-1 pathway, given its central role in the disease's pathogenesis. IL-1 inhibitors, such as anakinra and canakinumab, have shown remarkable efficacy in providing rapid and sustained relief from symptoms [10-12]. However, management can be challenging, especially in cases refractory to IL-1 inhibition or where diagnosis is delayed [13,14].

## Case presentation

A 40-year-old male presented with a six-month history of significant weight loss (28 pounds) and a persistent cough. For the past few weeks, he has been experiencing an urticarial rash across different body regions and joint pain (which was intermittent in nature and moderate in severity) in the bilateral knee and ankle joints. Initially, the pain was relieved by over-the-counter drugs but later became unresponsive to them. The patient's medical history was unremarkable. His family history included pancreatic cancer in his grandmother and breast cancer in his mother. The patient denied recent travel, smoking cigarettes, or using illicit drugs, though he occasionally used marijuana.

A physical examination revealed an urticarial rash on the upper and lower back and upper extremities and bilateral diffuse lymphadenopathy. Initial evaluation was significant for an elevated erythrocyte sedimentation rate (ESR) of 65 mm/hr, C-reactive protein (CRP) of 95 mg/dL (Table 1), cryoglobulin levels of 1200 mg/dL, and antinuclear antibody (ANA) titers of 1:160 (Table 2). The subsequent rheumatological assessment demonstrated negative results for anti-glomerular basement membrane (anti-GBM), anti-Sjögren's-syndrome-related antigen A (anti-SSA), anti-Sjögren's-syndrome-related antigen B (anti-SSB), cytoplasmic antineutrophil cytoplasmic antibodies (c-ANCA), perinuclear antineutrophil cytoplasmic antibodies (p-ANCA), and antiphospholipid antibodies (APLA). Viral serology for HIV, hepatitis B and C came back negative. The patient's chronic urticarial rash, arthralgias, and elevated inflammatory markers raised the possibility of Schnitzler syndrome, which prompts further work-up.

**Table 1:** Lab reports ESR - erythrocyte sedimentation rate; CRP - C-reactive protein

Lab reports	Results	Reference range
White blood cell count (1000/cumm)	12.4	4-11
Platelet count (1000/cumm)	189	150-450
Red blood cell count (million/uL)	3.89	4.5-5.1
Hemoglobin (g/dL)	11.5	14-16
Mean corpuscular volume (fL)	78.4	80-100
Mean corpuscular hemoglobin (pg)	27.5	27.5-33.2
Mean corpuscular hemoglobin concentration (gm/dL)	33.4	33.4-35.5
Neutrophils (%)	85	40-80
Lymphocytes (%)	10	20-40
Eosinophils (%)	1	1-6
Monocytes (%)	4	2-10
Basophils (%)	0	0-1
ESR (mm/hr)	43	0-20
CRP (mg/dL)	95	<0.3

**Table 2:** Miscellaneous lab reports

Miscellaneous	Results	Reference range
Anti-nuclear antibody	>1:160	<1:40
Anti-double-stranded DNA (IU/mL)	22	0-25
C3 (mg/dL)	95	90-150
C4 (mg/dL)	20	15-45
IgE Antibodies (mg/dL)	40	<0.3
Tryptase (ng/mL)	5	1-15
Cryoglobulin levels (mg/L)	1200	100-300

**Figure 1:**
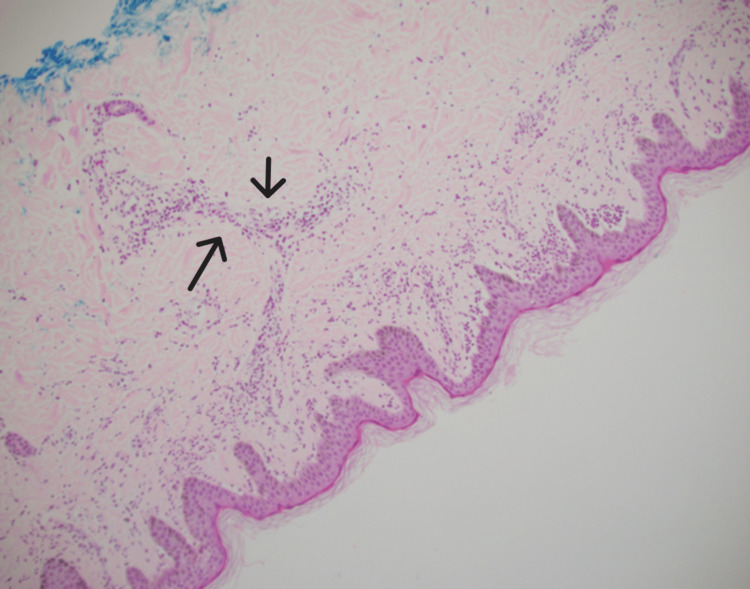
Perivascular and interstitial neutrophilic infiltrate within the superficial dermis

**Figure 2:**
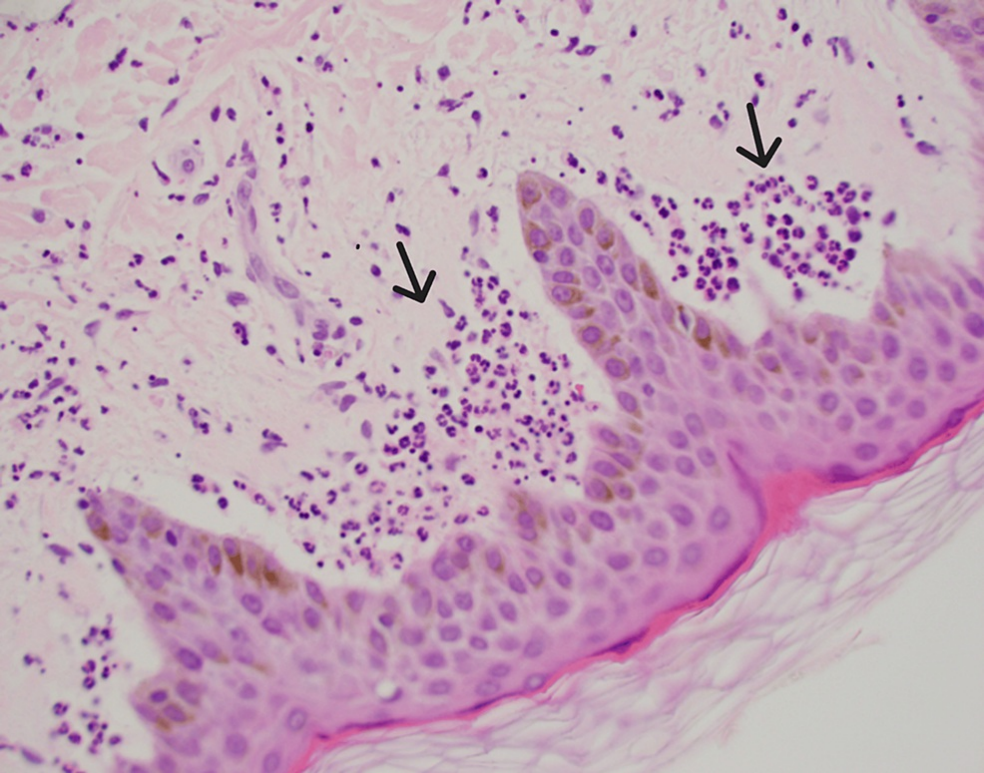
Papillary dermal neutrophilic micro abscesses

Further evaluation with CT scans of the chest, abdomen, and pelvis with contrast showed subpectoral, axillary, and inguinal lymphadenopathy. Fine-needle aspiration cytology (FNAC) of the left axillary lymph node was negative for malignancy and lymphoproliferative disorders. Bone marrow biopsy and aspiration showed a small cluster of kappa monotypic plasma cell population involving 2-3% of the bone marrow cells. Histopathological examination of skin biopsies revealed sparse perivascular and interstitial neutrophilic infiltrates with weak granular IgA deposition at the dermal-epidermal junction (Figures 1, 2). Despite the absence of IgM monoclonal protein, the presence of IgG kappa monoclonal gammopathy and a small plasma cell population on bone marrow biopsy (2-3% of cells) suggested a Schnitzler-like syndrome.

Based on the Strasbourg criteria for Schnitzler syndrome, which include a chronic urticarial rash (an obligatory criterion) and at least two minor criteria (recurrent fever, abnormal bone remodeling, elevated CRP/ESR, or monoclonal IgM component), the patient's presentation met the obligatory criterion and several minor criteria, justifying the diagnosis of Schnitzler-like syndrome.

The patient was initially treated with high-dose prednisone, providing symptomatic relief. A slow taper was implemented, and hydroxychloroquine was administered for ongoing rash management. Follow-up visits indicated a partial response to hydroxychloroquine, with significant improvement in fever and joint pain, although the urticarial rash persisted intermittently. Due to the insufficient response to prednisolone and hydroxychloroquine, the IL-1 inhibitor anakinra 100 mg subcutaneously daily was started. At the one-month follow-up, the patient responded well to anakinra.

## Discussion

This case underscores the diagnostic complexities and therapeutic challenges of Schnitzler-like syndrome with IgG kappa monoclonal gammopathy. A comprehensive diagnostic approach, including a detailed histopathological examination, is essential. The potential benefits of IL-1 inhibition in managing refractory cases are highlighted, along with the need for further research into the pathogenesis of Schnitzler-like syndrome and the establishment of standardized treatment protocols.

Differentiating Schnitzler-like syndrome from other autoinflammatory and hematologic disorders is complex. An extensive workup, including imaging and laboratory tests, is essential to exclude infections, malignancies, and other systemic inflammatory diseases. Elevated ESR and ANA, alongside IgG kappa monoclonal gammopathy, point towards an underlying inflammatory and possibly autoimmune process [8,9]. Histopathological examination of skin biopsies revealed neutrophilic dermatosis, seen in conditions like Schnitzler syndrome, adult-onset Still's disease, and cryopyrin-associated periodic syndromes [3].

According to the Strasbourg criteria for Schnitzler syndrome, our patient met the obligatory criterion of chronic urticarial rash and additional minor criteria, including recurrent fever, elevated ESR, and the presence of IgG kappa monoclonal gammopathy. This application of the criteria strengthens the diagnosis of Schnitzler-like syndrome.

Previous case reports, such as that by Bonnekoh et al. (2019), documented a case of Schnitzler-like syndrome with IgG kappa monoclonal gammopathy successfully treated with tocilizumab, an IL-6 inhibitor, suggesting the variability in cytokine involvement and treatment responses [8]. Similarly, Rensink et al. (2014) highlighted the importance of distinguishing Schnitzler syndrome from other periodic fever syndromes, given the overlapping symptoms and different treatment protocols [9]. These comparisons underscore the necessity of a thorough differential diagnosis in cases presenting with atypical gammopathy and systemic inflammatory symptoms.

The pathogenesis of Schnitzler syndrome involves IL-1-mediated inflammation, a key cytokine in the inflammatory cascade. IL-1 inhibitors, such as anakinra, have shown remarkable efficacy in providing rapid and sustained relief from symptoms, highlighting the central role of IL-1 in this disorder. The patient's partial response to hydroxychloroquine underscores the potential benefits of antimalarial drugs in managing autoinflammatory conditions, although IL-1 inhibitors remain the cornerstone of therapy for refractory cases [7,13].

In a multicenter study by Calvo-Río et al. (2014), IL-1 inhibitors demonstrated significant efficacy in treating Schnitzler syndrome, with most patients achieving complete remission of symptoms [10]. Additionally, Besada and Nossent (2010) reported dramatic improvements in a patient with longstanding multidrug-resistant Schnitzler syndrome treated with an IL-1 receptor antagonist, further supporting the effectiveness of IL-1 blockade [2].

The generalized lymphadenopathy and bilateral renal lesions observed in our patient warranted a thorough evaluation to rule out lymphoproliferative disorders and other malignancies. Fine-needle aspiration cytology and a bone marrow biopsy were critical in this assessment. The identification of IgG kappa monoclonal gammopathy and the small cluster of kappa monotypic plasma cells involving 2-3% of bone marrow cells indicated MGUS, a premalignant condition requiring regular monitoring to detect potential progression to multiple myeloma or other lymphoproliferative disorders [2,14].

Kyle and Rajkumar (2006) provided a comprehensive review of the epidemiology of plasma-cell disorders, emphasizing the importance of monitoring patients with MGUS for potential progression to multiple myeloma, reinforcing the need for vigilance in our patient's follow-up [14].

Future research should focus on elucidating the pathogenesis of Schnitzler-like syndrome, particularly the role of different monoclonal gammopathies. Understanding the genetic and molecular mechanisms underlying this condition could inform the development of targeted therapies and improve diagnostic accuracy. Long-term studies are needed to assess the risk of progression to malignancies in patients with Schnitzler-like syndrome and to establish standardized monitoring protocols.

Lipsker (2010) emphasized the necessity for further studies to understand the pathogenesis of Schnitzler syndrome, particularly the genetic factors contributing to its development [5]. Braud and Lipsker (2024) highlighted recent advances in understanding the clinical manifestations and management of Schnitzler syndrome, pointing towards a need for more specific and targeted therapeutic strategies [3].

## Conclusions

Schnitzler-like syndrome with IgG kappa monoclonal gammopathy is an uncommon and complicated disorder that requires early detection and treatment to improve patient outcomes and prevent the development of more serious conditions. The difficulty in identifying this condition originates from its overlapping symptoms with other autoinflammatory and hematologic illnesses, emphasizing the necessity for a thorough diagnostic strategy.

This case report contributes to the small but growing body of research on Schnitzler-like illness with IgG monoclonal gammopathy, emphasizing the need for thorough clinical and laboratory assessments in obtaining an appropriate diagnosis. Furthermore, the potential advantages of IL-1 inhibition in controlling refractory symptoms are highlighted, resulting in a focused therapy strategy that might greatly enhance patient quality of life. Future research should concentrate on better understanding the pathophysiology of this variation and creating uniform treatment procedures to improve clinical management.
